# Accelerated Epigenetic Age Among Women with Invasive Cervical Cancer and HIV-Infection in Nigeria

**DOI:** 10.3389/fpubh.2022.834800

**Published:** 2022-04-29

**Authors:** Jonah Musa, Kyeezu Kim, Yinan Zheng, Yishu Qu, Brian T. Joyce, Jun Wang, Drew R. Nannini, Demirkan B. Gursel, Olugbenga Silas, Fatimah B. Abdulkareem, Godwin Imade, Alani S. Akanmu, Jian-Jun Wei, Masha Kocherginsky, Kwang-Youn A. Kim, Firas Wehbe, Chad J. Achenbach, Rose Anorlu, Melissa A. Simon, Atiene Sagay, Folasade T. Ogunsola, Robert L. Murphy, Lifang Hou

**Affiliations:** ^1^Department of Preventive Medicine, Division of Cancer Epidemiology and Prevention, Northwestern University Feinberg School of Medicine, Chicago, IL, United States; ^2^Center for Global Oncology, Institute for Global Health, Northwestern University Feinberg School of Medicine, Chicago, IL, United States; ^3^Department of Obstetrics and Gynecology, College of Health Sciences, University of Jos, Jos, Nigeria; ^4^Department of Pathology, Northwestern University Feinberg School of Medicine, Chicago, IL, United States; ^5^Department of Pathology, University of Jos, Jos, Nigeria; ^6^Department of Pathology, College of Medicine, University of Lagos, Lagos, Nigeria; ^7^Department of Hematology and Blood Transfusion, Lagos University Teaching Hospital and College of Medicine, University of Lagos, Lagos, Nigeria; ^8^Department of Preventive Medicine, Division of Health and Biomedical Informatics, Northwestern University Feinberg School of Medicine, Chicago, IL, United States; ^9^Department of Obstetrics and Gynecology, College of Medicine, University of Lagos, Lagos, Nigeria; ^10^Department of Obstetrics and Gynecology, Northwestern University Feinberg School of Medicine, Chicago, IL, United States; ^11^Department of Medical Microbiology, College of Medicine, University of Lagos, Lagos, Nigeria

**Keywords:** invasive cervical cancer, human immunodeficiency virus, epigenetic age acceleration, DNA methylation, LMIC

## Abstract

**Background:**

Invasive cervical cancer (ICC) is a serious public health burden in Nigeria, where human immunodeficiency virus (HIV) remains highly prevalent. Previous research suggested that epigenetic age acceleration (EAA) could play a role in detection of HIV-associated ICC. However, little research has been conducted on this topic in Africa where the population is most severely affected by HIV-associated ICC. Here, we investigated the association between ICC and EAA using cervical tissues of ICC-diagnosed Nigerian women living with HIV.

**Methods:**

We included 116 cervical tissue samples from three groups of Nigerian women in this study: (1) HIV+/ICC+ (*n* = 39); (2) HIV+/ICC- (*n* = 53); and (3) HIV-/ICC + (*n* = 24). We utilized four DNA methylation-based EAA estimators; IEAA, EEAA, GrimAA, and PhenoAA. We compared EAA measurements across the 3 HIV/ICC groups using multiple linear regression models. We also compared EAA between 26 tumor tissues and their surrounding normal tissues using paired *t*-tests. We additionally performed a receiver operating characteristics (ROC) curve analysis to illustrate the area under the curve (AUC) of EAA in ICC.

**Results:**

We found the most striking associations between HIV/ICC status and PhenoAge acceleration (PhenoAA). Among HIV-positive women, PhenoAA was on average 13.4 years higher in women with ICC compared to cancer-free women (*P* = 0.005). PhenoAA was 20.7 and 7.1 years higher in tumor tissues compared to surrounding normal tissues among HIV-positive women (*P* = 0.009) and HIV-negative women (*P* = 0.284), respectively. We did not find substantial differences in PhenoAA between HIV-positive and HIV-negative women with ICC.

**Conclusion:**

PhenoAA is associated with ICC in HIV-infected women in our study. Our findings suggest that PhenoAA may serve as a potential biomarker for further risk stratification of HIV-associated ICC in Nigeria and similar resource-constrained settings.

## Introduction

Invasive cervical cancer (ICC), though preventable, continues to be a large burden and cause of cancer-mortality worldwide (1). In Nigeria, the mortality related to ICC is 22.9 per 100,000, with more than 9,600 deaths per year (2). Limited access to healthcare (3–5), as well as late detection and treatment, have contributed to the poor prognosis and high mortality for ICC-diagnosed women in Nigeria and most low- and middle-income countries (LMICs). As an acquired immunodeficiency syndrome (AIDS)-defining cancer, ICC is a more serious public health burden for LMICs in West Africa, including Nigeria (2, 6), where human immunodeficiency virus (HIV) remains highly prevalent (7).

HIV and its impact on immune dysfunction stimulate transmission and reactivation of oncogenic viral co-infections such as human papilloma virus (HPV). Previous studies suggested HIV-infected women were still vulnerable to onset of ICC despite of taking antiretroviral therapy (ART) (8, 9). Earlier research have proposed that molecular genetic factors, such as immunogenetic variations, play a role in progression of cervical cancer among HIV/HPV co-infected women (10, 11). However, the molecular mechanisms by which long-term HIV infection may promote cervical carcinogenesis remains largely unknown, although recent evidence suggest that HIV infection may cause accelerated molecular aging (12, 13).

Epigenetic age acceleration (EAA) is one potential DNA methylation-based biomarker of cancer. On the basis of accumulated age-related changes in DNA methylation, EAA estimators were developed to predict biological aging, healthspan, and lifespan (14–17). Several EAA estimators have been proposed to date including intrinsic epigenetic age acceleration (IEAA), extrinsic epigenetic age acceleration (EEAA), acceleration of Levine's phenotypic age (PhenoAA), and acceleration of GrimAge (GrimAA) (14–17). Although the EAA measurements have been originally designed to measure biological aging and predict lifespan, they have shown strong associations with age-related health outcomes including cancer (16–20). Evidence highlighting the associations between EAA and various types of cancer shows its promise in cancer detection (18, 20–22). To our best knowledge, most studies evaluated EAA in blood, which raised the necessity of studies exploring EAA with tissues in cancer origin (23).

Studies focused on the association between EAA and ICC are scarce although using EAA could be promising based on the previous evidence which demonstrated the associations of epigenetic changes including DNA methylation with the initiation, development, and progression of cervical cancer (24–27). Furthermore, while sub-Saharan Africa has a high burden of HIV and associated ICC, most studies on epigenetic biomarkers have been conducted outside of Africa, limiting the potential application of EAA to ICC screening in this region. Previous research found accelerated epigenetic age among individuals with HIV, which implies that HIV infection prompts molecular aging (12, 13). Taken together, it is important to explore EAA in individuals with coinfection of HIV and ICC to expand current understanding; however, little is done in this area of study.

In this study, we investigated the associations between ICC and the EAA measures described above among ICC-diagnosed women living with HIV in Nigeria. Our aim for this study is to understand the potential application and utility of EAA as a biomarker for screening and early detection of cervical precancer in a setting with high burden of HIV and ICC.

## Methods

### Study Participants and Data Collection

Eligible study participants were recruited from two federal academic tertiary hospitals in Nigeria, Jos University Teaching Hospital (UniJos) and the Lagos University Teaching Hospital (UniLag), in 2018–2020. Trained recruiters who worked alongside the clinical care teams at UniJos and UniLag determined participant eligibility by reviewing clinical appointment lists. Study enrollment criteria included women aged 18 or older who were not pregnant, had no history of hysterectomy, and were not receiving cervical cancer treatment at the time of recruitment. For the purpose of the study to compare the variations in EAA by ICC status among women living with HIV, HIV-positve participants were classified by their ICC status: HIV-positive women with ICC (study group; HIV+/ICC+) and HIV-positive women without ICC (control group 1: HIV-/ICC+). To further investigate whether the variations in EAA are upon by the status of HIV, we added another control group (control group 2: HIV+/ICC-). Participants' clinical and demographic information were obtained by interviewer-administered questionnaire at the time of enrollment into the study. The intent and procedures of the study were introduced to the study participants, and informed consent was obtained. The study protocol was reviewed and approved by the Institutional Review Boards (IRBs) at UniJos, UniLag, and Northwestern University in Chicago, Illinois, USA.

### Definition of HIV Infection

The HIV status of study participants receiving care and treatment at the Presidential Emergency Plan for AIDS Relief (PEPFAR) program of the two participating institutions was extracted from the adult HIV treatment and care database. Women with unknown HIV status had rapid HIV diagnostic testing according to the national HIV testing serial algorithm, which involved the use of the Rapid Determine Test, Unigold, and STAT Pack rapid HIV diagnostic test kits. All HIV-infected women receiving care in the PEPFAR program at both study sites were on antiretroviral therapy (ART) at the time of study enrollment. Those whose HIV infection was diagnosed at enrollment were provided appropriate HIV counseling and linked to care and commencement of ART in the PEPFAR program of the participating institutions.

### Diagnosis of Invasive Cervical Cancer

Suspected ICC cases were evaluated by the oncology team of study investigators at UniJos and UniLag. Diagnostic evaluation of ICC included examination under anesthesia, international federation of gynecology and obstetrics (FIGO 2009 or 2018 versions) clinical staging, and cervical tissue biopsy for histopathological diagnosis and tumor grading by trained pathologists at the two enrollment institutions with quality control and telepathology review by the Northwestern's Pathology core. The clinical staging of ICC was determined in accordance with the International Federation of Gynecology and Obstetrics (FIGO) staging system. Cancer grades were categorized as: well-differentiated (grade 1), moderately differentiated (grade 2), and poorly differentiated (grade 3). All histopathological diagnoses were independently done by certified pathologists at UniJos and UniLag. The histopathology slides and paraffin-fixed blocks were shared with the Northwestern University Cancer Center Pathology Core team for verification. Cervical tumor was determined as the primary tumor for the participants with ICC in this study.

### Cervical Tissue DNA Extraction and Quantification

Approximately 25–30 ng of tumor and normal cervical tissue obtained during the biopsies were used for tissue extraction following laboratory protocols developed by trained laboratory personnel at the two Nigerian study sites in collaboration with the Pathogenomic Core at Northwestern University. QIAGEN QIAamp DNA Mini Kit and Quibit 4 techniques were used for DNA extraction/purification and quantification, respectively. Samples with DNA concentration of 500ng and above were shipped to Northwestern University for DNA methylation profiling using the EPIC assay.

### DNA Methylation Profiling and Calculation of EAA

Profiling of DNA methylation was performed using the participants' cervical tissue. In addition to the DNA obtained from 121 tumor and normal tissue samples, we also assayed 29 surrounding normal tissue samples from ICC patients. We generated Infinium MethylationEPIC BeadChip raw data using the cervical tissue samples. R packages *minfi* (28) and *ENmix* (29) were used to load the raw data and perform quality control, respectively. We excluded 8,896 CpGs with a low detection rate (detection rate <0.95) and one sample with poor methylation quality levels or low intensity of bisulfite conversion probes (<3 standard deviations from the mean). Two outlier samples were also excluded based on Tukey's method (30). The remaining samples went through preprocessing procedures using the R package *minfi* (28), using the *preprocessIllumina* function. Two surrounding normal tissue samples from ICC patients were removed as their paired tumor tissue did not pass the methylation data quality check. The QC procedure and data preprocessing resulted in a total of 142 analytic samples, with 116 individual samples (63 tumor and 53 normal samples) and 26 surrounding normal tissue samples paired with tumor samples from the ICC patients.

We calculated 4 epigenetic age measurements, Horvath's DNAm age, Hannum's age, PhenoAge, and GrimAge, using Horvath's DNA Methylation Age Calculator (https://dnamage.genetics.ucla.edu), based on the published algorithms (14–17). Acceleration of each epigenetic age measurement, IEAA, EEAA, PhenoAA, and GrimAA, was then computed as the regression residuals of each epigenetic age against chronological age, representing the independent deviation of epigenetic age from chronological age. A positive EAA represents a higher-than-expected epigenetic age relative to chronological age and vice versa.

### Final Analytic Data and Statistical Analysis

We combined phenotype data and epigenetic age measurements from the DNA methylation data to obtain the final analytic data (*n* = 116, for individual samples). Paired samples (*n* = 52, 26 normal tissues and 26 tumor tissues) were analyzed separately in this study. We explored the demographic and clinical characteristics of study participants using *t*-tests and Chi-squared tests. We assessed EAA across the three HIV/ICC groups using multiple regressions, comparing the study group (HIV+/ICC+) to each control group (HIV+/ICC- and HIV-/ICC+, respectively). Confounders such as participants' education level, cancer stage, tumor grade, body mass index (BMI), and study site were included in the models as covariates. We also included eight control probe principal components (PCs) in the models to account for batch and technical bias (explaining 86.4% of variation). Chronological age was not included in the models as a covariate as EAA measurements were already adjusted for chronological age in the main analysis models (14–17). We investigated the correlations of EAA with cancer stage and tumor grade, to further evaluate the difference EAA by cancer stage and tumor grade.

Among the women with ICC, we compared EAA between 26 tumor tissues and their surrounding normal tissues using paired *t*-tests, stratified by HIV status. We also conducted a receiver operating characteristics (ROC) curve analysis to illustrate the area under the curve (AUC) of EAA in ICC. All associations with *P* < 0.05 were declared significant. SAS version 9.4 (SAS Institute, Cary, NC) was used for all statistical analysis.

## Results

Table 1 presents the demographic characteristics of study participants. Women with HIV+/ICC+ tended to be older at the time of study enrollment, younger at the time of their first pregnancy, and have more live births. Participants' BMI and employment status were comparable across all three groups. Among women with ICC, there was no remarkable difference in cancer stage at study enrollment. We also investigated the correlation of EAA measurements with cancer stage and tumor grade among women with ICC, however we did not observe any differences by cancer subgroups (data not shown).

**Table 1:** Characteristics of study participants by HIV and ICC status.

		**HIV + /ICC+**	**HIV + /ICC-**	**HIV-/ICC +**	***p***
		**(*N* = 39)**	**(*N* = 53)**	**(*N* = 24)**	
Age, mean (SD)		54.2 (11.4)	43.8 (9.1)	53.0 (13.9)	<0.001
Age at first pregnancy, mean (SD)		19.5 (2.6)	22.7 (6.2)	22.0 (2.6)	0.008
BMI, mean (SD)		29.3 (9.3)	26.4 (5.9)	25.5 (4.5)	0.081
Employment, *N* (%)	Employed	28 (71.8)	41 (77.4)	19 (79.2)	0.911
	Unemployed	10 (25.6)	11 (20.8)	5 (20.8)	
	Missing	1 (2.6)	1 (1.9)	0 (0.0)	
Education, *N* (%)	Less than primary	20 (52.6)	18 (34.0)	13 (54.2)	0.113
	Secondary	6 (15.8)	22 (41.5)	6 (25.0)	
	Tertiary	12 (30.8)	13 (24.5)	5 (20.8)	
	Missing	1 (2.6)	0 (0.0)	0 (0.0)	
Parity, *N* (%)	0–5 livebirths	16 (41.0)	46 (86.8)	13 (54.2)	<0.001
	6+ livebirths	20 (51.3)	4 (7.6)	11 (45.8)	
	Missing	3 (7.7)	3 (5.7)	0 (0.0)	
Cancer stage, *N* (%)	Stage I/II	19 (49.0)	NA	16 (66.7)	0.197
	Stage III/IV	19 (49.0)	NA	8 (33.3)	
	Missing	1 (2.0)	NA	0 (0.0)	
Tumor grade, *N* (%)	Grade 1	8 (20.5)	NA	2 (8.3)	0.028
	Grade 2	18 (46.2)	NA	14 (58.3)	
	Grade 3	1 (2.6)	NA	5 (20.8)	
	Missing	12 (30.8)	NA	3 (12.5)	
HIV treatment, *N* (%)	Received	7 (18.0)	46 (86.8)	NA	<0.001
	Not received	4 (10.3)	0 (0.0)	NA	
	Missing	28 (71.8)	7 (13.2)	NA	
Study site. *N* (%)	Lagos	1 (2.6)	20 (38.5)	23 (100.0)	<0.001
	Jos	38 (97.4)	32 (61.5)	0 (0.0)	

Table 2 shows the associations between ICC status and EAA among HIV-positive women. Of the four EAA measurements, we found the most striking associations between ICC status and PhenoAA among HIV-positive women. Among HIV-positive women, PhenoAA among women with ICC was 16.2 years higher than cancer free women (95% CI = 9.8, 22.6, *P* < 0.001) in the crude model. After further adjusting for education, BMI, methylation principal components, and study site, PhenoAA among women with ICC was 13.4 years higher than cancer free women (95% CI = 3.9, 22.9, *P* = 0.005). The other two EAA measurements, GrimAA and EEAA, were also higher among women with ICC than cancer free women (5.4 years for GrimAA and 1.6 years for EEAA, respectively) whereas IEAA among women with ICC were 9.2 years lower than cancer free women. However, no associations between the three EAA measurements (GrimAA, EEAA, and IEAA) and ICC status persisted in fully adjusted models.

**Table 2:** Associations between ICC status and EAA measurements among HIV positive participants (HIV + /ICC + vs. HIV + /ICC-; *N* = 92).

**EAA**	**Model 1**		**Model 2**	
	**Beta (95% CI)**	***P*-value**	**Beta (95% CI)**	***P*-value**
PhenoAA	16.2 (9.8, 22.6)	<0.001	13.4 (3.9, 22.9)	0.005
GrimAA	5.4 (2.5, 8.2)	<0.001	1.1 (−3.3, 5.5)	0.616
IEAA	−9.2 (−13.8, −4.5)	<0.001	−2.8 (−9.7, 4.2)	0.433
EEAA	1.6 (−3.6, 6.9)	0.544	5.1 (−2.5, 12.6)	0.188

*The unit of measurement is in years; Model 1: crude model; Model 2: adjusted for education, BMI at enrollment, control probe PCs, and study site; for comparison of HIV+/ICC+ vs. HIV-/ICC+, cancer stage and tumor grade were additionally adjusted in the model*.

Table 3 presents the associations between HIV status and EAA among ICC diagnosed women. Unlike the comparison of EAA by ICC status, we did not find substantial differences in PhenoAA by HIV status although HIV-positive women showed 5.3 years higher PhenoAA after adjusting for covariates (95% CI: −30.1, 40.7, *P* = 0.770). Similarly, we did not observe discrepancies in other EAA measurements by HIV status.

**Table 3:** Associations between HIV status and EAA measurements among ICC participants (HIV+/ICC+ vs. HIV-/ICC+; *N* = 63).

**EAA**	**Model 1**		**Model 2**	
	**Beta (95% CI)**	***P*-value**	**Beta (95% CI)**	***P*-value**
PhenoAA	2.5 (−8.6, 13.5)	0.661	5.3 (−30.1, 40.7)	0.770
GrimAA	0.2 (−4.2, 4.6)	0.931	4.6 (−10.2, 19.5)	0.540
IEAA	−3.9 (−11.6, 3.6)	0.306	−23.3 (−49.1, 2.5)	0.077
EEAA	−3.2 (−11.4, 5.0)	0.445	23.5 (−4.8, 51.9)	0.104

*The unit of measurement is in years; Model 1: crude model; Model 2: adjusted for education, BMI at enrollment, control probe PCs, cancer stage, tumor grade, and study site*.

Table 4 presents the difference in EAA from paired samples in women with ICC by HIV status. Among HIV+/ICC+ women, PhenoAA was 20.7 years higher in tumor tissues compared to the surrounding normal tissues (95% CI = 6.3, 35.1, *P* = 0.009). Similarly, we observed GrimAA was 7.9 years higher in tumor tissues compared to the surrounding normal tissues (95% CI: 1.4, 14.4, *P* = 0.021). The other two EAA measurements, IEAA and EEAA, did not present substantial differences in tumor tissues compared to surrounding normal tissues. We also did not observe substantial differences in all four EAA measurements from tumor tissues comparing their surrounding normal tissues among HIV-/ICC+ women.

**Table 4:** Comparison of EAA in normal and tumor tissues among women with ICC.

**EAA**	**Tissue type**	**HIV+/ICC+ women (*N* = 39)**			**HIV-/ICC+ women (*N* = 24)**		
		**Mean (SD)**	**Mean.diff^†^ (95% CI)**	***P*-value**	**Mean (SD)**	**Mean.diff^†^ (95% CI)**	***P*-value**
PhenoAA	Tumor	15.4 (21.5)	20.7 (6.3, 35.1)	0.009	7.9 (21.3)	7.1 (−6.6, 20.7)	0.284
	Normal	−5.3 (8.9)			0.8 (10.4)		
GrimAA	Tumor	3.0 (8.0)	7.9 (1.4, 14.4)	0.021	3.5 (8.5)	2.9 (−2.8, 8.7)	0.295
	Normal	−5.0 (7.0)			0.6 (6.7)		
IEAA	Tumor	−1.9 (11.7)	−4.9 (−12.2, 2.4)	0.164	−0.9 (18.1)	0.5 (−8.8, 9.8)	0.902
	Normal	3.0 (7.6)			−1.5 (15.1)		
EEAA	Tumor	−4.0 (15.3)	−3.6 (−12.2, 4.9)	0.365	1.9 (17.2)	−2.0 (−9.1, 5.1)	0.552
	Normal	−0.4 (9.7)			3.9 (9.1)		

*The unit of measurement is in years;*

† *Difference of mean EAA between tumor tissue and surrounding normal tissue*.

**Figure 1:**
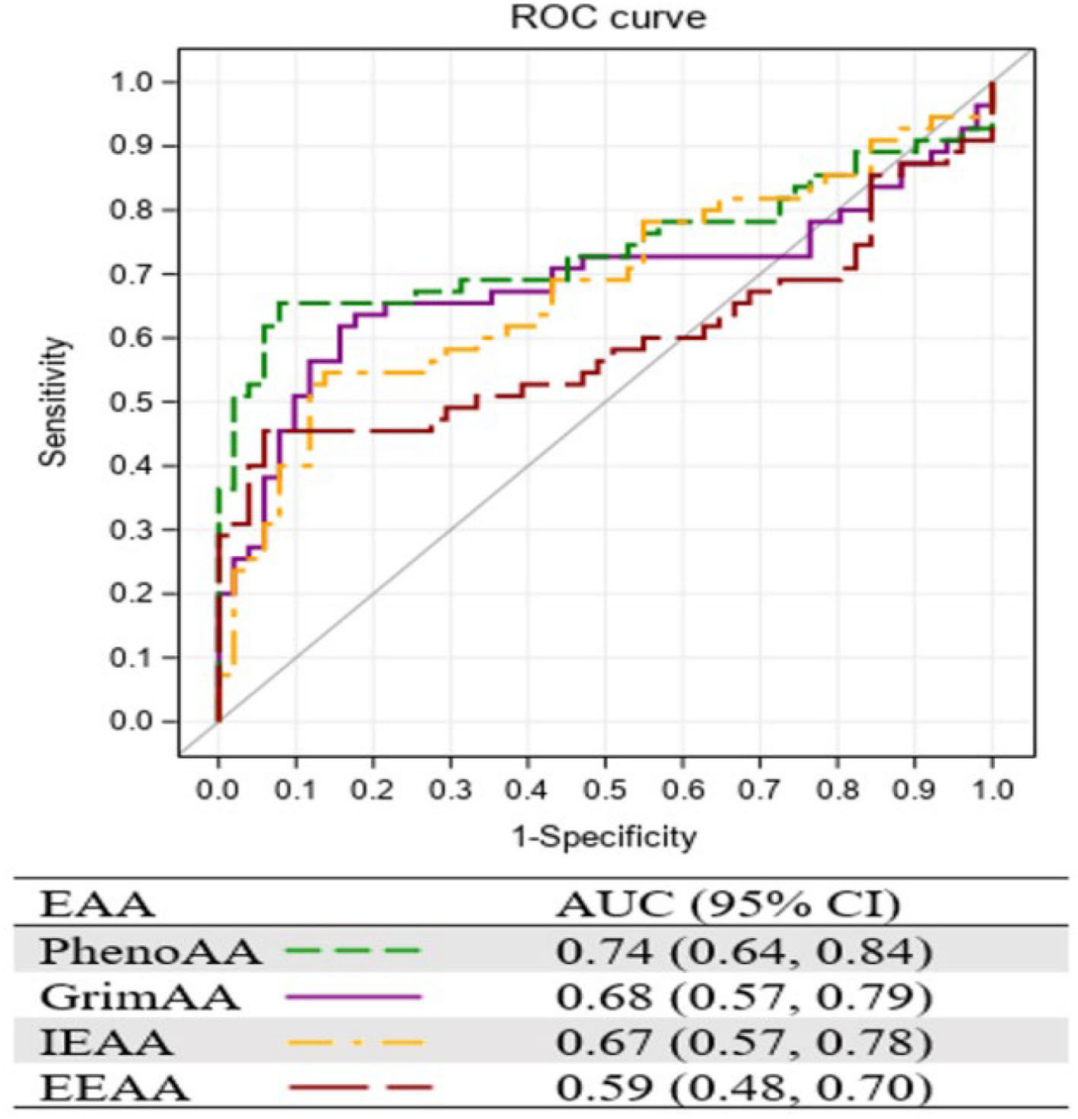
Area under the curve (AUC) of 4 EAA measurements from ROC curve analysis.

Figure 1 presents the AUC of the four EAA measurements resulting from the ROC curve analysis. Consistent with the strongest association with ICC, PhenoAA showed the greatest AUC (0.74, 95% CI: 0.64, 0.84) among all EAA measurements included in the study, followed by GrimAA (AUC = 0.68, 95% CI: 0.57, 0.79).

## Discussion

In this study, we assessed EAA among women with ICC and HIV in Nigeria. We observed the greatest PhenoAA among women with both ICC and HIV (HIV+/ICC+), compared to HIV+/ICC- and HIV-/ICC+ women. Among HIV+/ICC+ women, we observed higher PhenoAA in tumor tissues compared to their surrounding normal tissues. We found stronger associations of ICC status with PhenoAA in comparison to HIV status. We also found the supportive AUC value with PhenoAA, which implies its potential usefulness.

Accelerated EAA among cancer patients in our study is in line with previous studies that reported greater EAA in cancer patients than controls (14, 18, 20, 22). Our findings of accelerated PhenoAge in tumor tissue also support prior evidence of greater deviation of epigenetic age from chronological age in tumor tissue among cervical cancer patients (31). Our findings are biologically plausible and in keeping with known mechanisms for cancer development. For instance, we know that epigenetic markers with age-associated DNA methylation accumulate over time and reflect alterations in transcriptional programs, and that tumor cells tend to present deviated methylation patterns with the age-associated CpGs, which may lead to carcinogenesis (32–34). Recent evidence shows genes with hyper-methylated age-associated CpGs in their promoters are involved in controlling cell development and signaling pathways (35, 36), showing substantial overlaps of CpGs with those used in PhenoAge calculation (16). Coupled with the evidence above, previously reported associations between PhenoAge components, including serum glucose (37, 38), albumin, and C-reactive protein (CRP) (39, 40), and cervical cancer also support the biological plausibility of our study. Prior studies have linked glucose metabolism to apoptosis in cervical cancer (37, 38). Albumin-CRP ratio is a known biomarker for cervical cancer progression (39, 40), which supports the association between ICC and PhenoAA in our study.

It is interesting to note that we did not observe differences in EAA by HIV status among ICC patients. Our findings differ from previous studies that reported substantially accelerated epigenetic age among individuals with HIV (12, 13); however, direct comparison of the results is limited as our study did not include disease-free individuals. The results from our study may suggest that ICC development has a larger effect on the epigenetic aging process than HIV infection alone. Another possibility is the effect of HIV treatment on DNA methylation. In our paired analysis, we observed lesser PhenoAA in the surrounding normal tissues of HIV+/ICC+ women, which was even lower than PhenoAA in the normal tissues among HIV-/ICC+ women. However, we did not have sufficient information on ART to reliably investigate the potential protective effect of HIV treatment on PhenoAA in the current study. The impact of HIV treatment on epigenetic alteration has been understudied, and we believe it should be a direction for future studies.

To our knowledge, this is the first study to explore the association of epigenetic aging biomarkers with HIV and ICC in Nigeria. The validation of this biomarker in subsequent studies could translate to more effective, easy and acceptable options for cervical cancer screening, particularly in LMICs, where there are several cultural, systemic and economic barriers for utilization of pap smears or HPV testing. While pap smears and HPV tests operated by healthcare provider raised concerns of cultural barriers, HPV tests using self-collected samples could lower them. However, HPV tests with self-collected samples also suffer with low specificity and high cost for a single test that would lead to multiple medical visit and increase of medical burden, which play a bigger role in LMICs (4, 41). As an alternative option, epigenetic biomarkers can be stably collected from various source of samples, and once collected, an epigenetic marker could be used for multiple purposes, including diagnosis and therapy, thus could be cost-effective (42). Coupled with the limited utilization of the existing methods in LMICs and the benefits from the use of epigenetic biomarkers, our results suggest that EAA may serve as a useful tool for risk stratification of ICC in the population with high HIV prevalence. To achieve the goal, future studies investigating the role of EAA using other types of samples (e.g., blood or saliva) are needed.

Our study was benefitted from examining cervical tissue samples to assess PhenoAge in ICC women, however it should be stated that PhenoAge was developed based on blood DNA-methylation levels. Nonetheless, PhenoAge showed a meaningful correlation with chronological age in normal cervical tissue, showing a correlation coefficient of 0.58 (16). This moderate correlation implies that greater deviation of PhenoAge from chronological age in cervical tissue, represented by greater PhenoAA, could be a proxy to detect the abnormal status of the cervix, including ICC. Furthermore, it would also be worthwhile to investigate the association of PhenoAA with ICC using other types of less invasive samples such as blood, self-collected cervicovaginal swabs, or urine. It could be promising, being supported by high concordance between self-collected urine and vaginal sampling and cervical sampling for detection of HPV (43, 44). We believe future studies are needed to examine the usefulness of PhenoAA in detecting ICC using those samples, especially considering the cultural barriers that limit the use of Pap smears and HPV tests in Nigeria and countries with similar cultural backgrounds.

Our study has several limitations. By study design, disease-free women who were HIV-negative and cancer-free were not included in our study, which precluded the ability to separate the effects of ICC and HIV on EAA. However, our results showed that the associations with EAA were stronger with ICC status rather than HIV status, which implies a future direction of the study area. The small sample size also limited the statistical power of our analysis which raised a caution for overinterpretation of the findings, and future studies with larger study populations, as well as different ethnic populations, are needed to expand the understanding of the current study. Finally, although we knew all HIV-infected women in this study were on ART at the time of enrollment, we did not have sufficient information on the biological markers of ART effects on virological suppression (viral load) or immunological status (CD4 counts). Treatment for HIV may be involved in the associations between disease status and PhenoAA, or may have a protective effect on PhenoAA itself; however, we were not able to control for this possibility in the current study. Additional studies are needed to unravel the interactive effects of components related to ART and ICC.

In conclusion, PhenoAA was positively associated with ICC status among HIV-infected women in Nigeria. To our knowledge, ours is the first epigenetic aging study of HIV-associated ICC in Africa. Using epigenetic age directly measured from cervical tissue, our study results more strongly support a role of epigenetic-related aging in HIV-associated ICC. We anticipate that our findings contribute to the evidence for potential utility of epigenetic aging markers, including PhenoAA, for cervical cancer screening and risk stratification of HIV-associated ICC in Nigeria and similar resource-constrained settings. Future study using more types of samples such as blood, saliva, or self-collected cervicovaginal swabs is needed for broader and more practical application of DNA methylation-based biomarkers in resource-constrained countries. We expect those efforts may lead to a practical and competitive screening method that mitigates the limitations of other existing screening methods. Thus we anticipate that PhenoAA could serve as a useful tool for screening and early detection of HIV-associated ICC in LMICs and, along with currently available screening tools (e.g., HPV testing), contribute to improved prevention and outcomes for cervical cancer.

## Data Availability Statement

The relevant data have been presented in the manuscript. Any required data could be provided by the corresponding authors on reasonable request.

## Ethics Statement

The studies involving human participants were reviewed and approved by Institutional Review Board at University of Lagos Institutional Review Board at University of Jos Institutional Review Board at Northwestern University. The patients/participants provided their written informed consent to participate in this study.

## Author Contributions

LH, RM, JM, YZ, MS, and CA conceptualized the study and developed the study protocols with support from the pathogenomic laboratory core (JW, DG, OS, FBA, GI, AA, and J-JW). MK, K-YK, and FW designed the database and managed project data and statistical support. JM and KK had full access to the data and led the writing of the manuscript. KK performed the statistical analysis and interpreted the results. YZ and YQ performed the DNA methylation preprocessing and epigenetic age calculation. JW led the laboratory process for DNA methylation profiling. RA, BJ, DN, AS, and FO provided additional support on data collection and interpretation of results. All the listed authors contributed in editing of the draft manuscript and approved the final version of the manuscript for submission.

## Funding

Research findings reported in this manuscript was supported by the National Cancer Institute of the National Institutes of Health under award number U54CA221205, and by the Fogarty International Center of the National Institutes of Health under award number D43TW009575. JM received funding through an International Research Career Development Award from the NIH/FIC (Grant #K43TW011416) that provided research-protected time for writing and review of this manuscript. The content is solely the responsibility of the authors and does not necessarily represent the official views of the National Institutes of Health.

## Conflict of Interest

The authors declare that the research was conducted in the absence of any commercial or financial relationships that could be construed as a potential conflict of interest.

## Publisher's Note

All claims expressed in this article are solely those of the authors and do not necessarily represent those of their affiliated organizations, or those of the publisher, the editors and the reviewers. Any product that may be evaluated in this article, or claim that may be made by its manufacturer, is not guaranteed or endorsed by the publisher.

## Abbreviations

AIDS: acquired immunodeficiency syndrome
EEAA: extrinsic epigenetic age acceleration
GrimAA: GrimAge acceleration
HIV: human immunodeficiency virus
HPV: human papilloma virus
ICC: Invasive cervical cancer
IEAA: intrinsic epigenetic age acceleration
LMICs: low- and middle income countries
PhenoAA: PhenoAge acceleration.
